# Nausea and vomiting during pregnancy associated with lower incidence of preterm births: the Japan Environment and Children’s Study (JECS)

**DOI:** 10.1186/s12884-018-1911-1

**Published:** 2018-06-27

**Authors:** Naomi Mitsuda, Masamitsu Eitoku, Keiko Yamasaki, Masahiko Sakaguchi, Kahoko Yasumitsu-Lovell, Nagamasa Maeda, Mikiya Fujieda, Narufumi Suganuma, Japan Environment & Children’s Study (JECS) Group, Toshihiro Kawamoto, Hirohisa Saito, Reiko Kishi, Nobuo Yaegashi, Koichi Hashimoto, Chisato Mori, Shuichi Ito, Zentaro Yamagata, Hidekuni Inadera, Michihiro Kamijima, Toshio Heike, Hiroyasu Iso, Masayuki Shima, Yasuaki Kawai, Narufumi Suganuma, Koichi Kusuhara, Takahiko Katoh

**Affiliations:** 1 Department of Environmental Medicine, Kochi Medical School, Kochi University, Kohasu, Oko-cho, Nankoku, Kochi 783-8505 Japan; 2 Integrated Center for Advanced Medical Technologies, Kochi Medical School, Kochi University, Kochi, Japan; 3 Department of Obstetrics and Gynecology, Kochi Medical School, Kochi University, Kochi, Japan; 4 Department of Pediatrics, Kochi Medical School, Kochi University, Kochi, Japan; 5 Cancer Prevention and Control Division, Kanagawa Cancer Center Research Institute, Kanagawa, Japan

**Keywords:** Nausea and vomiting during pregnancy, Preterm birth, JECS

## Abstract

**Background:**

Nausea and vomiting during pregnancy (NVP) is considered to be associated with favorable fetal outcomes, such as a decreased risk for spontaneous abortion. However, the relationship between NVP and preterm births remains unknown. This study was conducted to evaluate the association between NVP and the risk of preterm births.

**Methods:**

The dataset of a birth cohort study, the Japan Environment and Children’s Study (JECS), was retrospectively reviewed. Participants’ experience of NVP prior to 12 gestational weeks were evaluated by a questionnaire administered from 22 weeks of pregnancy to 1 month before delivery. NVP responses were elicited against four choices based on which the study population was divided into four subcohorts. Preterm birth was the main study outcome. Logistic regression analysis was used to quantify an association between NVP and risk of preterm birth.

**Results:**

Of 96,056 women, 79,460 (82.7%) experienced some symptoms of NVP and 10,518 (10.9%) experienced severe NVP. Compared to those who did not experience NVP, women with severe NVP had lower odds for preterm birth [adjusted odds ratio (aOR) 0.84, 95% confidence interval (95% CI) 0.74–0.95]. An even lower OR was found among very preterm birth and extremely preterm birth (aOR 0.44, 95% CI 0.29–0.65).

**Conclusion:**

An inverse association exists between NVP and preterm births, especially, very preterm births and extremely preterm births.

## Background

Nausea and vomiting during pregnancy (NVP) is among the most common clinical complaints in the first trimester of pregnancy. NVP affects up to 70% of pregnant women, but there is considerable variation among reported frequencies (35–91%) [1]. NVP has been posited to have multifactorial causation, including genetic, endocrine, and gastrointestinal factors [2–4]. However, a clear etiopathogenesis of NVP has not been established. Some studies have shown that NVP represents a favorable hormonal milieu, accompanied by larger placentas and elevated levels of chorionic gonadotrophin and estrogens in pregnant women [5, 6]. Another hypothesis asserts that the role of NVP is to protect pregnant women and embryos from foodborne pathogens and dietary toxins [7, 8].

In the context of these hypotheses, NVP is associated with favorable fetal outcomes [9, 10]. A number of past studies linked NVP with a decreased risk for spontaneous abortion [11–14]. Some studies have shown that women who experience NVP have a lower risk of preterm birth than those without such symptoms [15, 16], although other studies have shown no association or reported the opposite [17–19]. This study was conducted to evaluate the association between maternal NVP and preterm birth from the data of the Japan Environment and Children’s Study (JECS).

## Methods

### Study design

We retrospectively analyzed the dataset of the JECS, a long-term birth cohort study to elucidate the influence of chemical exposures during the fetal period and early childhood on children’s health with follow-up until age 13. The protocol and baseline data of this study are available elsewhere [18].

For the JECS, pregnant women were recruited between January 31, 2011 and March 31, 2014. Eligibility criteria for study participants (expectant mothers) were as follows: 1) residing in the study areas at the time of recruitment and attending collaborating healthcare providers; 2) expected delivery date after August 1, 2011; and 3) capable of comprehending Japanese and completing self-administered questionnaires. Details of the JECS project have been described in a previous article [20].

With regards to exposure measurement, lifestyle and other background information was collected using a self-administered questionnaire distributed to participating pregnant women from the first trimester up to 21 weeks and 6 days of pregnancy (M-T1) and from 22 weeks of pregnancy to 1 month before delivery (M-T2). Medical histories of past and present pregnancies, and physical status of participants and their offspring, were collected from an obstetrician’s medical chart at registration (Dr-T1) and at delivery (Dr-0 m). Study analyses were based on M-T2, Dr-T1, and Dr-0 m.

### Sample selection

**Fig. 1:**
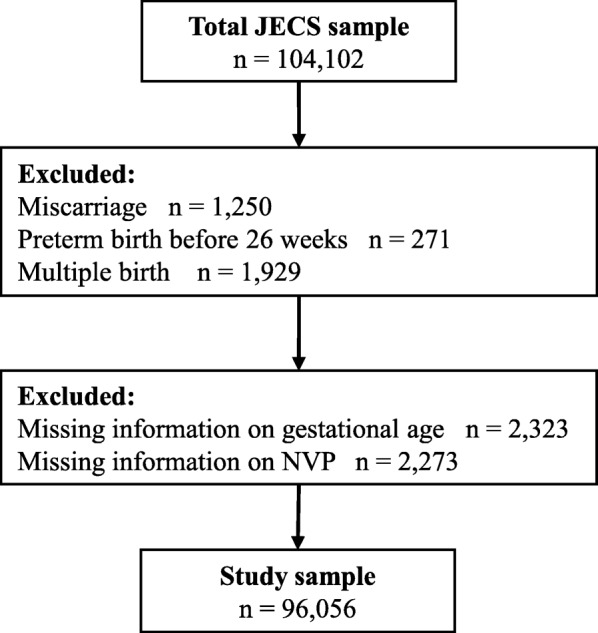
Flowchart for Selection of Participants from JECS

The present study was based on the “jecs-ag-20,160,424”, which was released in June, 2016. The JECS dataset included 104,102 births. We excluded miscarriage (*n* = 1250) and multiple births (*n* = 1929). We also excluded women who had delivered before 26 weeks of gestation because they may have delivered before the M-T2 questionnaire was provided (*n* = 271). Moreover, we excluded cases with missing data on gestational age (*n* = 2323), and cases with missing data on NVP (*n* = 2273). In total, 96,056 births were included in the final study sample (Fig. 1).

### Variables

Information on NVP, maternal education, and maternal smoking habits during pregnancy were obtained from M-T2. In M-T2, participants were asked whether they experienced NVP in the first 12 gestational weeks (1. did not experience NVP; 2. nausea only; 3. experienced NVP but could have meals; and 4. experienced NVP and could not have meals).

Information on parity, maternal height, and pre-pregnancy weight was obtained from Dr-T1, and data on maternal age, gestational age, birth outcomes, and prenatal complications were obtained from Dr-0 m. Participants underwent ultrasound examinations during the first trimester, and these results were used to determine the expected date of delivery if there was more than a 7-day difference between this date and the date calculated from the last menstrual period.

Maternal age was categorized into six groups: younger than 20 years, 20–24 years, 25–29 years, 30–34 years, 35–39 years, and 40 years or older. By data on parity, the cohort was classified into nullipara and multipara. A proxy for socioeconomic status, maternal length of education, was categorized into ≤12 years and > 12 years. Body mass index (BMI) was calculated from the information on pre-pregnancy height and weight and categorized into three groups: underweight (< 18.5 kg/m^2^), normal (18.5–24.9 kg/m^2^) and overweight (≥25 kg/m^2^). Maternal smoking habits were categorized into smoking during pregnancy and others.

Gestational age, defined as an outcome variable, was categorized as post term (≥42 weeks), term (37–41 weeks) and preterm (< 37 weeks). Further, preterm birth was subdivided into moderately preterm (32–36 weeks), very preterm (28–31 weeks), and extremely preterm (26–27 weeks) [21].

### Statistical analyses

The study population was divided into four groups, based on the answers to the questionnaire for NVP symptoms as follows: those who did not experience NVP (no NVP); those who experienced nausea only (mild NVP); those who experienced NVP but could have meals (moderate NVP); and those who experienced NVP and could not have meals (severe NVP). Maternal characteristics and pregnancy outcomes were compared among the four NVP groups. Gestational age was compared employing Kruskal–Wallis test for non-normally distributed data. Categorical variables were compared using a chi-squared test. *P*-values < 0.05 indicated statistical significance.

Logistic regression analysis was used to estimate the association between NVP and the risk of preterm birth. For analysis of the odds ratio (OR) of preterm birth, we used a dichotomized outcome variable: preterm birth (< 37 weeks) and others (≥37 weeks). For analysis of OR of very preterm birth and extremely preterm birth, we used another dichotomized outcome variable: very preterm birth and extremely preterm birth (born at < 32 weeks of gestation) and others (born at ≥32 weeks). ORs were adjusted for maternal age, parity, maternal education, maternal pre-pregnancy BMI, and smoking habits during pregnancy. Results are presented as crude odds ratios (cOR) and adjusted odds ratios (aOR), or as mean differences with 95% confidence intervals (95% CI). All analyses were conducted using Stata 13.1 (Stata Corp, Texas).

## Results

As shown in Table 1, the 96,056 pregnant women were categorized into no NVP (*n* = 16,596; 17.3%), mild NVP (*n* = 41,198; 42.9%), moderate NVP (*n* = 27,744; 28.9%), and severe NVP (*n* = 10,518; 10.9%). Higher rates of no symptoms of NVP were seen among women with older age, nullipara, higher education, low pre-pregnancy BMI, and smoking during pregnancy.

**Table 1:** Maternal characteristics according to NVP status

	Total	No NVP	Mild NVP	Moderate NVP	Severe NVP	*P* value
	*n* = 96,056	*n* = 16,596	*n* = 41,198	*n* = 27,744	*n* = 10,518	
	n	n (%)	n (%)	n (%)	n (%)	
Maternal Age (years)						
< 20	814	190 (1.2)	256 (0.6)	256 (0.9)	112 (1.1)	< 0.001
20–24	8630	1678 (10.1)	3026 (7.4)	2787 (10.1)	1139 (10.8)	
25–29	26,528	4487 (27.0)	10,775 (26.2)	8111 (29.2)	3155 (30.0)	
30–34	34,075	5432 (32.7)	14,997 (36.4)	9967 (35.9)	3679 (35.0)	
35–39	21,622	3861 (23.3)	10,038 (24.4)	5636 (20.3)	2087 (19.8)	
≥ 40	4383	945 (5.7)	2106 (5.1)	986 (3.6)	346 (3.3)	
Missing^a^	4					
Parity						
Nullipara	37,800	8244 (51.4)	15,404 (38.2)	10,040 (36.9)	4112 (40.1)	< 0.001
Multipara	56,005	7804 (48.6)	24,875 (61.8)	17,170 (63.1)	6156 (60.0)	
Missing^a^	2251					
Education (years)						
≤ 12	34,790	6398 (38.7)	14,118 (34.4)	10,320 (37.3)	3954 (37.8)	< 0.001
> 12	60,893	10,126 (61.3)	26,931 (65.6)	17,315 (62.7)	6521 (62.3)	
Missing^a^	373					
BMI (kg/m^2^)						
< 18.5	15,500	2859 (17.2)	6834 (16.6)	4167 (15.0)	1640 (15.6)	< 0.001
18.5–24.9	70,221	12,141 (73.2)	30,216 (73.4)	20,351 (73.4)	7513 (71.5)	
≥ 25	10,272	1584 (9.6)	4122 (10.0)	3209 (11.6)	1357 (12.9)	
Missing^a^	63					
Smoking during pregnancy						
No	90,887	15,325 (93.3)	39,155 (95.8)	26,294 (95.5)	10,113 (97.0)	< 0.001
Yes	4386	1106 (6.7)	1724 (4.2)	1239 (4.5)	317 (3.0)	
Missing^a^	783					
Pregnancy Complications						
Threatened abortion	11,428	1695 (10.2)	4885 (11.9)	3451 (12.4)	1397 (13.3)	< 0.001
Threatened premature labor	18,715	2896 (17.5)	7990 (19.4)	5566 (20.1)	2263 (21.5)	< 0.001
Premature rupture of membrane	7932	1549 (9.3)	3374 (8.2)	2218 (8.0)	791 (7.5)	< 0.001
Pregnancy-induced hypertension	2964	622 (3.8)	1189 (2.9)	841 (3.0)	312 (3.0)	< 0.001

Chi-squared test

^a^ Not included in percentage distribution

The prevalence of pregnancy-related complications possibly causing preterm birth were also significantly different among the four groups. In women without NVP, the prevalence of threatened abortion and threatened premature labor were lowest, whereas rates of preterm rupture of membrane and pregnancy-induced hypertension were highest (Table 1).

The overall rate of preterm births (< 37 weeks) was 4.6% (4397/96,056). Rates of extremely (26–27 weeks), very (28–31 weeks), and moderately (32–36 weeks) preterm births were 0.09% (88/96,056), 0.38% (364/96,056), and 4.1% (3929/96,056), respectively. Median gestational age was not statistically influenced by NVP status. However, the prevalence of preterm birth was slightly higher in women without NVP (Table 2). When compared to women without NVP, women with mild or moderate NVP had lower odds for overall preterm births (aOR 0.87, 95% CI 0.80–0.95 and aOR 0.85, 95% CI 0.78–0.93, respectively), and women with severe NVP had the lowest odds (aOR 0.84, 95% CI 0.74–0.95; Table 3). Differences between women with and without NVP were more obvious when the risk of very preterm birth and extremely preterm birth was analyzed. When compared to women without NVP, women with mild or moderate NVP had lower odds for very preterm birth and extremely preterm birth (aOR 0.74, 95% CI 0.58–0.94 and aOR 0.62, 95% CI 0.47–0.82, respectively), and women with severe NVP had the lowest odds (aOR 0.44, 95% CI 0.29–0.67; Table 4).

**Table 2:** Distribution of gestational week according to NVP status

	Total	No NVP	Mild NVP	Moderate NVP	Severe NVP	*P* value
	*n* = 96,056	*n* = 16,596	*n* = 41,198	*n* = 27,744	*n* = 10,518	
Gestational week, Median (p5–p95), weeks						
	39.4 (37.0–41.1)	39.4 (36.9–41.3)	39.4 (37.0–41.1)	39.4 (37.0–41.1)	39.4 (37.0–41.3)	0.006^a^
Gestational week, n(%)						
26–27 week	88	16 (0.10)	46 (0.11)	20 (0.07)	6 (0.06)	< 0.001^b^
28–31 week	364	94 (0.6)	152 (0.4)	93 (0.3)	25 (0.2)	
32–36 week	3929	743 (4.5)	1674 (4.1)	1089 (3.9)	423 (4.0)	
37–41 week	91,454	15,694 (94.6)	39,224 (95.2)	26,495 (95.5)	10,041 (95.5)	

^a^ Kruskal-Wallis test

^b^ Chi-squared test

**Table 3:** Odds ratio of preterm birth in relation to NVP status

	No NVP	Mild NVP			Moderate NVP			Severe NVP		
		OR	95% CI	*P* value	OR	95% CI	*P* value	OR	95% CI	*P* value
Crude Odds ratio	1(reference)	0.88	0.81–0.95	0.002	0.84	0.76–0.91	< 0.001	0.83	0.74–0.94	0.002
Adjusted Odds ratio^a^	1(reference)	0.87	0.80–0.95	0.002	0.85	0.78–0.93	0.001	0.84	0.74–0.95	0.004

^a^ Adjusted for maternal age, BMI, smoking, education and parity

**Table 4:** Odds ratio of very preterm birth and extremely preterm birth in relation to NVP status

	No NVP	Mild NVP			Moderate NVP			Severe NVP		
		OR	95% CI	*P* value	OR	95% CI	*P* value	OR	95% CI	*P* value
Crude Odds ratio	1(reference)	0.72	0.57–0.91	0.007	0.61	0.47–0.80	< 0.001	0.44	0.30–0.66	< 0.001
Adjusted Odds ratio^a^	1(reference)	0.74	0.58–0.94	0.01	0.62	0.47–0.82	0.001	0.44	0.29–0.67	< 0.001

^a^ Adjusted for maternal age, BMI, smoking, education and parity

## Discussion

In our nationwide cohort study of approximately 100,000 births, we found that NVP symptoms were associated with decreased risk of preterm birth. An even lower OR was found for very preterm birth and extremely preterm birth. Furthermore, pregnancy complications such as preterm rupture of membrane and pregnancy-induced hypertension were less frequent in women who experienced at least some symptoms of NVP than in women with no NVP.

These findings are similar to the results of a Norwegian large cohort study showing higher prevalence of preterm births in women who did not experience NVP than in women who did experience NVP [15]. Czeizel showed that women who had medically recorded NVP and were treated for it had longer gestational age and a lower proportion of preterm birth than women who had mild NVP without any treatment or hospitalization due to hyperemesis gravidarum [16]. Klebanoff reported lower rates of preterm births in women who reported vomiting during pregnancy [13]. These two results are also similar to our findings, whereas Naumann and Weigel reported no association between NVP and rate of preterm births [18, 19], and Temming reported higher rates of preterm births in women who reported NVP [17].

As Czeizel indicated, these previous inconsistent results may be attributed to the difference in the definition/classification of NVP [16]. Various classifications of NVP were used in past studies because NVP still has no universally accepted definition/classification. Some studies classified NVP into three categories: no NVP, nausea only, and nausea/vomiting [14, 15, 19]. Other studies classified NVP into two categories, but how they assigned the categories varied [13, 16, 18]. Koren established a scoring system for NVP, classifying them into none, mild, moderate, and severe according to the number of vomiting or retching episodes and the length of nausea episodes [6, 22]. In our study, although the questionnaire about NVP had four choices, it did not collect information on duration or frequency of NVP. Therefore, we might not be able to precisely estimate the severity of NVP from our questionnaire in the manner Koren recommended. However, when we analyzed the variables of NVP by merging three choices with any symptoms of NVP into one category, the association between NVP and preterm births remained negative (data not shown).

Other possible reasons for previous inconsistent results may be the mild effect of NVP on gestational age and the difference of sample size [16]. In this study, the association between NVP and decreased risk of preterm birth was statistically significant. However, the clinical impact induced by this association is considered to be modest. Therefore, a large sample size is needed to demonstrate the association between NVP and birth outcomes. Previous studies which demonstrated an association between NVP and decreased risk of preterm birth had a large sample size. For example, Czeizel examined 38,151women and Chortatos examined 51,675 women, respectively [15, 16]. However, studies which concluded there was no association between NVP and preterm birth had smaller sample sizes [17, 18]. Our study showed the generalizability of previous findings from large cohort studies to an Asian population.

The etiopathogenesis of NVP, although unclear, is likely to be multifactorial, including placenta-mediated mechanisms [5]. Niebyl mentioned that NVP is less common in older women, multiparous women and smokers, which is attributed to the smaller placental volumes in these women [23]. In our study, NVP was less common in older women and smokers, as in Niebyl’s study. Furthermore, NVP is more common in women with high BMI. This fact further supports Niebyl’s speculation, because overweight and obese women generally have a heavier placenta [24]. However, multiparous women experienced NVP more often than nulliparous women in this study, which diverges from Niebyl’s report. To prove the hypothesis that placental volume affects symptoms of NVP, future investigations on the association between placental characteristics and NVP status is needed.

Several limitations pertaining to this study should be considered. Information on the duration of NVP, treatment of NVP and late onset NVP could not be obtained. Therefore, we could not assess prolonged NVP and late onset NVP. The fact that information on maternal characteristics and NVP were missing in some cases was also a limitation. Some women who delivered preterm babies before 26 weeks may have failed to answer the questionnaire because they may have delivered before the questionnaire was provided. Therefore, we excluded women who had delivered before 26 weeks of gestation.

The strengths of our study are that it is a large population-based cohort study. To our knowledge, our study is the largest study to date on this topic and the first study to evaluate the association between NVP and preterm births in the Asian population. These data enabled us to analyze risks of subgroups that experienced preterm birth against a range of confounding factors, including maternal characteristics and prenatal risk factors. The fact that information on NVP were obtained before delivery is another strength of this study. Besides the adjustment for confounders, the standardized healthcare system in Japan and the relatively homogeneous Japanese pregnant population should limit possibilities of residual confounding.

## Conclusion

NVP was inversely associated with preterm births, especially for very preterm births and extremely preterm births. Further investigation of the association between severity of NVP and placental characteristics or hormonal milieu is needed.

## Abbreviations

aOR: Adjusted odds ratio
BMI: Body mass index
CI: Confidence interval
cOR: Crude odds ratio
JECS: The Japan Environment and Children’s Study
NVP: Nausea and vomiting during pregnancy
OR: Odds ratio

## References

[1] Einarson TR, Piwko C, Koren G (2013). Quantifying the global rates of nausea and vomiting of pregnancy: a meta analysis. J Popul Ther Clin Pharmacol.

[2] Colodro-Conde L, Cross SM, Lind PA, Painter JN, Gunst A, Jern P, Johansson A, Lund Maegbaek M, Munk-Olsen T, Nyholt DR, et al. Cohort Profile: Nausea and vomiting during pregnancy genetics consortium (NVP Genetics Consortium). Int J Epidemiol. 2017;46(2):e17. 10.1093/ije/dyv360 PMC5837614 26921609

[3] Lagiou P, Tamimi R, Mucci LA, Trichopoulos D, Adami HO, Hsieh CC (2003). Nausea and vomiting in pregnancy in relation to prolactin, estrogens, and progesterone: a prospective study. Obstet Gynecol.

[4] Masson GM, Anthony F, Chau E (1985). Serum chorionic gonadotrophin (hCG), schwangerschaftsprotein 1 (SP1), progesterone and oestradiol levels in patients with nausea and vomiting in early pregnancy. Br J Obstet Gynaecol.

[5] Bustos M, Venkataramanan R, Caritis S (2017). Nausea and vomiting of pregnancy - What's new?. Auton Neurosci.

[6] Lacasse A, Rey E, Ferreira E, Morin C, Berard A (2008). Validity of a modified pregnancy-unique quantification of Emesis and nausea (PUQE) scoring index to assess severity of nausea and vomiting of pregnancy. Am J Obstet Gynecol.

[7] Sherman PW, Flaxman SM (2002). Nausea and vomiting of pregnancy in an evolutionary perspective. Am J Obstet Gynecol.

[8] Flaxman SM, Sherman PW (2008). Morning sickness: adaptive cause or nonadaptive consequence of embryo viability?. Am Nat.

[9] Huxley RR (2000). Nausea and vomiting in early pregnancy: its role in placental development. Obstet Gynecol.

[10] Koren G, Madjunkova S, Maltepe C (2014). The protective effects of nausea and vomiting of pregnancy against adverse fetal outcome--a systematic review. Reprod Toxicol.

[11] Hinkle SN, Mumford SL, Grantz KL, Silver RM, Mitchell EM, Sjaarda LA, Radin RG, Perkins NJ, Galai N, Schisterman EF (2016). Association of Nausea and Vomiting during Pregnancy with Pregnancy Loss: a secondary analysis of a randomized clinical trial. JAMA Intern Med.

[12] Chan RL, Olshan AF, Savitz DA, Herring AH, Daniels JL, Peterson HB, Martin SL (2010). Severity and duration of nausea and vomiting symptoms in pregnancy and spontaneous abortion. Hum Reprod.

[13] Klebanoff MA, Koslowe PA, Kaslow R, Rhoads GG (1985). Epidemiology of vomiting in early pregnancy. Obstet Gynecol.

[14] Weigel MM, Weigel RM (1989). Nausea and vomiting of early pregnancy and pregnancy outcome. An epidemiological study. Br J Obstet Gynaecol.

[15] Chortatos A, Haugen M, Iversen PO, Vikanes A, Eberhard-Gran M, Bjelland EK, Magnus P, Veierod MB (2015). Pregnancy complications and birth outcomes among women experiencing nausea only or nausea and vomiting during pregnancy in the Norwegian mother and child cohort study. BMC Pregnancy Childbirth.

[16] Czeizel AE, Puho E (2004). Association between severe nausea and vomiting in pregnancy and lower rate of preterm births. Paediatr Perinat Epidemiol.

[17] Temming L, Franco A, Istwan N, Rhea D, Desch C, Stanziano G, Joy S (2014). Adverse pregnancy outcomes in women with nausea and vomiting of pregnancy. J Matern Fetal Neonatal Med.

[18] Naumann CR, Zelig C, Napolitano PG, Ko CW (2012). Nausea, vomiting, and heartburn in pregnancy: a prospective look at risk, treatment, and outcome. J Matern Fetal Neonatal Med.

[19] Weigel MM, Reyes M, Caiza ME, Tello N, Castro NP, Cespedes S, Duchicela S, Betancourt M (2006). Is the nausea and vomiting of early pregnancy really feto-protective?. J Perinat Med.

[20] Kawamoto T, Nitta H, Murata K, Toda E, Tsukamoto N, Hasegawa M, Yamagata Z, Kayama F, Kishi R, Ohya Y (2014). Rationale and study design of the Japan environment and children's study (JECS). BMC Public Health.

[21] Tucker J, McGuire W (2004). Epidemiology of preterm birth. BMJ.

[22] Koren G, Boskovic R, Hard M, Maltepe C, Navioz Y, Einarson A (2002). Motherisk—PUQE (pregnancy-unique quantification of emesis and nausea) scoring system for nausea and vomiting of pregnancy. Am J Obstet Gynecol.

[23] Niebyl JR (2010). Clinical practice. Nausea and vomiting in pregnancy. N Engl J Med.

[24] Wallace JM, Horgan GW, Bhattacharya S (2012). Placental weight and efficiency in relation to maternal body mass index and the risk of pregnancy complications in women delivering singleton babies. Placenta.

